# Smart Monochromatic Composite: A Literature Review

**DOI:** 10.1155/2022/2445394

**Published:** 2022-11-08

**Authors:** Muhammad Adeel Ahmed, Rizwan Jouhar, Zohaib Khurshid

**Affiliations:** ^1^Department of Restorative Dental Sciences, College of Dentistry, King Faisal University, Al-Ahsa 31982, Saudi Arabia; ^2^Department of Prosthodontics and Dental Implantology, College of Dentistry, King Faisal University, Al-Ahsa 31982, Saudi Arabia

## Abstract

Over the previous years of the 20^th^ and 21^st^ centuries, there has been a progression in the field of bonded esthetic restorations. At the present time, the inclination of dentists toward the smart monochromatic shade of composite is flourishing owing to the fact that it decreases the requirement of a range of composite shades, curtails the waste of unconsumed composite shades, lessens chair side interval, abolishes the shade selection, and decreases dependency on shade-selecting methods. Smart monochromatic composite is known to obtain the color of the adjacent tooth structure in which it is placed. Therefore, the current literature elucidates the several features of innovative and revolutionary monochromatic composites including color stability, mechanical and optical properties, and shade-matching capability that could have a positive impact potentially over other resin composites.

## 1. Introduction

In the last century, restorative dentistry has presented much development in resin composite including adhesive technology and techniques. Previously, acrylic resins were used as a restorative material; however they had several disadvantages: such as poor abrasion resistance, low color stability, higher shrinkage, and poor peripheral seal [1]. Consequently, R. Bowen introduced polymeric restorations that were reinforced with quartz filler that recognized as “resin composite.” Composite is a three-dimensional compound which consists of two or more chemically dissimilar materials with excellent properties than those of an individual component. Resin composite presents extremely conservative and esthetic restorations to an individual owing to significant progression along with its compatible use for the last couple of decades. Formerly, composites were suggested as a restorative material merely for anterior teeth; however at present, fillers combined with acid etching and its good compatibility to tooth structure made it worthwhile for both anterior and posterior restorations [2]. Presently, resin composites are recommended as an inexpensive and esthetic substitute to other direct and indirect restorations in consequence of its optimization of formulations, up gradation of properties, and innovative methods for application [3].

### 1.1. History of Resin Composite

#### 1.1.1. Macrofilled Composites

In the early 1970s, macrofilled composites were introduced. The leading commercial composite resins were {Concise (3 M) and Adaptic (Dentsply Sirona)}. They are composed of large fillers with typical particle sizes ranging from 0 to 5 *μ*m with rough surface texture. Wearing off occlusal contact area with deposition of plaque occurs due to hardness of filler particles. Their physical and mechanical properties are superior over unfilled acrylic resins. They are recommended in pressure-bearing areas, for instance, Classes I and II and large size cavities of Classes III and IV [4].

#### 1.1.2. Microfilled Composites

In the 1980s, microfilled composites were developed. The commercially popular resin composites were Durafill VS (Kulzer) and Renamel (Cosmedent) with usual particle size ranging from 0.04 to 0.4 *μ*m [5]. They have a polished and smooth surface texture because of small particle size that enables resin composite to resist against plaque, debris, and stain. They carried inferior mechanical properties owing to higher matrix content, lower color stability, and increased marginal breakdown. They are indicated as restoration of anterior teeth and cervical lesions [5].

#### 1.1.3. Hybrid Composites

In the 1990s, hybrid composites were introduced. As reflected by name, they are composed of organic part which is reinforced by an inorganic phase [6]. They were difficult to polish because different sizes of glasses were used in their composition, with particle size of <2 *μ*m and comprise 0.04 *μ*m-sized fumed silica as well. They exhibited admirable polishing and texturing properties, better abrasion and wear resistance, and reduced polymerization shrinkage. They presented higher surface smoothness and better strength recommended for both anterior and posterior restorations. Consequently, in the era of 2000, innovative formulations were introduced with improved esthetic properties. This was the first-time that variations in shades have been permitted to emulate the natural tooth structure.

#### 1.1.4. Nanofilled and Nanohybrid Composites

After the year of 2000, nanofilled and nanohybrid composites were developed as Tetric EvoCeram (Ivoclar Vivadent) and Filtek Supreme Plus (3 M) with typical particle size ranging from 5 to 75 nm and nanocluster fillers with particle size ranging from 5 to 20 nm that were less than that of microfilled composites [6, 7]. They exhibited better physical properties similar to the original hybrid resin composite and restorations with a smoother surface texture and polish [8].

**Figure 1:**
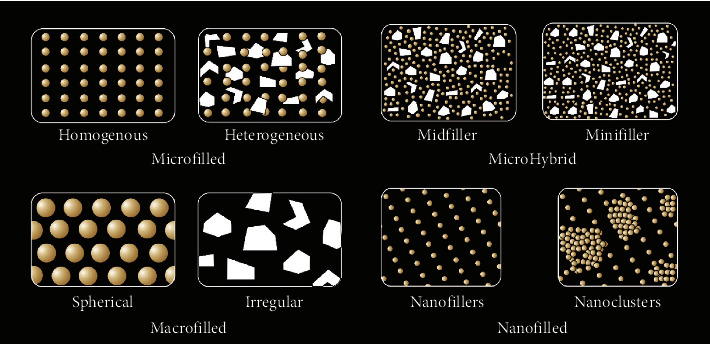
Classification of the composite on the basis of particle size and structure.

Classification of the composite on the basis of particle size and structure is shown in Figure 1.

#### 1.1.5. Bulk-Fill Composites

By the 2010s, bulk-fill composites were introduced which got approval by many dental practitioners due to less significant polymerization shrinkage with a better depth of cure up to 4 mm [8]. The first flowable bulk-fill composite was recognized as SureFil SDR Flow (Dentsply Sirona) that was applied as a base beneath restorations. Newer bulk-fill agents such as Tetric EvoCeram Bulk-Fill (Ivoclar Vivadent) and Estelite Bulk Flow (Tokuyama Dental America) do not need any additional layer of composite as a crowning. They revealed greater strength and better esthetics, but some of them were translucent that showed their advantages and disadvantages reliant on the restorations [9].

One research revealed that pigments in food or drinks or habits such as smoking cause extrinsic or intrinsic staining of composites. Thus, bleaching techniques are applied to have desired esthetic. Bleaching can eliminate developed stains on composites and can reproduce their original shade; however, it cannot modify the shade of composite restorations to a brighter color. Because of this reason, bleaching is commonly suggested before restoration of an anterior composite so that composite restoration is harmonized to the original and brighter tooth shade [10].

### 1.2. The Contemporary Change

#### 1.2.1. Smart Monochromatic Composite

Smart monochromatic composite is a leading shade-matching composite that gained more acceptance in recent times. It possesses distinctive characteristics that are based on “smart chromatic technology.” It has the capability to capture the structural color of its surrounding tooth that is controlled by the size of its filler particles [11]. It has no extra dyes or pigments, whereas fillers itself produce red-to-yellow structural color that matches the surrounding tooth color. Color is the light wavelength that enters into our eyes. Human teeth come into the range of red-to-yellow color [11]. Smart monochromatic composite is a one-shade material that is specified to match entirely 16 VITA Classical shades (VITA North America, Yorba Linda, CA). It has another shade that is opaque, termed as Blocker to represent the color of dentine in translucent areas like restorations in class IV cavities.

Smart monochromatic composite has a distinctive feature that helps clinicians not be confused by many shades. It presents a rapid and easy method that makes striking and functionally esthetic restorations. Smart monochromatic composite has been recognized to possibly save time in the clinic to get rid of the requirement of shade selection. In this composite, material has homogeneously sized spherical-shaped filler particles. It adjusts the light that is transmitted all along the red-to-yellow area of the color scale and shows matching the color of neighboring teeth of patients [11].

The main characteristics of smart monochromatic composite include better polishing capability, superior flexural and compressive strength, easy handling, clinically satisfactory outcomes, and resistant to ambient light. It carries minimal wear of composite and opposing tooth structure. Smart monochromatic composite is available in the form of opaque-white paste that allows the material more visible to clinicians during manipulation and placement. The material is evenly mixed with adjacent teeth prior to application of light source during curing. A chamfered margin is preferred to get better marginal seal [12].

A single shade is only required to match color in Classes I and II restoration in posterior teeth. In case of extensive Class III and Class IV restorations of anterior teeth, a blocking agent could be applied as 0.5 mm thin coat prior to insertion of the smart monochromatic composite. Particularly, in case of discoloration, it camouflages the internal portion of the crown. Additionally, it also reduces the shade-matching interference [11]. To the best of our knowledge, various brands of smart monochromatic composites available in the market to date are summarized in Table 1.

## 2. Literature Review

### 2.1. Color-Generating Phenomena

**Figure 2:**
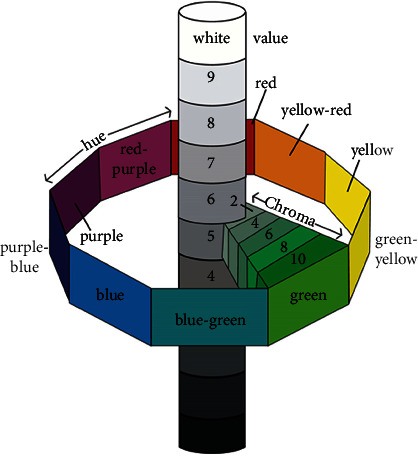
Munsell color system (adapted and redrawn from ©1994 Encyclopedia Britannica, Inc.).

Munsell sphere shows the wide-ranging perceptible color space (Figure 2). The natural color teeth range is relatively restricted and distributed in A narrow range of red to yellow from A1 to D4, indicating variable grades of darkness, lightness, and saturation [13]. Teeth color matching is related to two color-producing phenomena:

#### 2.1.1. Chemical Color

Chemical color is the common form of perceptible color that results as material particles reflect specific wavelengths. Chemical color is produced by adding dyes and pigments that most commonly present in various composites. Currently, most composites need many shades to mimic every dental shade. They rely on red and yellow colorants that are added to the resin material to match tooth shades [14].

#### 2.1.2. Structural Color

Structural color occurs while different light wavelengths are augmented or declined by the material structure itself showing colors other than what the material may actually be. It rarely exists, and the effects can be spectacular, for instance, colors in nature, from morphs of butterflies to peacocks, in addition to soap bubble film and surfaces of compact disc. Smart monochromatic composite uses structural color mechanism in composite dentistry, without dyes or pigments. The ideal match from A1 to D4 and beyond is produced by the spherical fillers itself that create red-to-yellow structural color that blends with the color of the neighboring dentition [11]. Smart monochromatic composite is composed of 260 nm spherical fillers that are the exact dimension and outline needed to create red-to-yellow color when available light crosses through the composite.

### 2.2. Components of the Smart Monochromatic Composite

- Fillers: Spherical-shaped identical in size supra-nano filler particles (260 nm SiO2-ZrO2) that are formed in regular edges. This gave an idea of development for polychromatic composite.
- Monomers: UDMA/TEGDMA with filler loading of 79 wt% (68 vol%). UDMA: urethane dimethacrylate and TEGDMA: triethyleneglycol dimethacrylate.

### 2.3. Recommendations for the Smart Monochromatic Composite

- Direct restorations in both posterior and anterior teeth.
- Direct composite veneering.
- Diastema closure or closure of space between any teeth.
- Composite and porcelain repair [11].

### 2.4. Key Characteristics of the Smart Monochromatic Composite

#### 2.4.1. Shade-Matching Ability

Harmonizing the shade of resin composite with anterior teeth is a difficult task that is experienced by the dental practitioners regularly. The color of underlying dentine has an effect on tooth shade [15]. Normally, various factors make the color matching challenging for dental practitioners. Color matching depends on different chromatic features that are associated with resin composite and teeth such as hue, chroma, and value; opalescence, translucency, and fluorescence; light diffusion and transmission; and surface texture properties [16, 17]. It is imperative for restorative material to imitate the natural tooth with all chromatic characteristics along with the color stability to have ideal esthetics [18].

Over the past decades, modifications have been executed to enhance the esthetic properties of resin composite restorative materials. Recently, single-shade structurally colored universal composites are directly applicable in most cases. They have the capability to change color according to the adjacent dentition. Consequently, they can enhance the appearance of the restoration esthetically as well as reduce the dependency on many shade-matching methods [19] and shade guide tabs [20, 21]. It was revealed that smart monochromatic material had no pigments and dyes, so its color properties are dependent solely on the physical properties of light. It has excellent color-matching ability for all shades [22, 23].

#### 2.4.2. Esthetic Properties and Effect of Bleaching on Surface Roughness

Resin composites are most accepted esthetic restorative materials that are used in dentistry due to their excellent optical properties, sufficient strength, and inherent bonding to tooth structure [24]. Another significant characteristic is a good surface texture without porosities that makes the restoration clinically successful because rough surfaces of restorations encourage plaque deposition, staining, and gingival irritation which eventually develops secondary caries [25]. Surface properties of composite restorations are affected by the oral environment and usual dietary habits that have a negative impact on the strength of composite restoration. In addition, few dental procedures, for instance, tooth bleaching, have a negative effect on resin composite filling materials, which is an easy and noninvasive method for tooth whitening based primarily on oxidation by hydrogen peroxide or one of its precursors [26]. Tooth bleaching causes undesirable alteration in resin composites as compared to other tooth-colored restorative materials because of the existence of organic matrix component. Bleaching agents that are used in bleaching treatment had peroxides that can provoke deterioration of the organic matrix complex of resin composites and cause surface roughness [27].

#### 2.4.3. Surface Texture and Color Stability

Surface smoothness and color stability are necessary for resin composite to be clinically successful. Multiple factors can affect the color stability of restorative material such as absorption of water, extent of polymerization, dietary habits of an individual, and surface irregularity of the restoration [28]. It is stated that the color sensitivity of material has a direct impact on polishing and finishing steps in addition to components of material [29]. Rough surface of the restoration becomes discolored by the effect of external factors such as coffee, tea, or red wine [30, 31]. Consequently, it is evidently supported that the smoothness of restoration enhances its esthetic appearance and success of restorative material, whereas surface roughness increases the probability of plaque deposition, secondary caries, and staining of the restoration [32]. Furthermore, it has also been found in the literature that the finishing and polishing system comprising diamond particles provides the least color difference on single-shade composite restorations [33, 34].

The color stability of a resin composite relates to organic matrix, magnitude of filler particles, polymerization depth, and coloring agents [35]. Similarly, another research by Kowalska et al. reported that the chemical variations in resin components like fraction of oligomers and monomers, proportion or kind of activators, initiators and inhibitors and oxidation of nonreactive carbon-carbon double bonds may have an influence on color stability [36]. Existence of micro cracks and micro voids at the merging point between the filler and the resin matrix are more susceptible areas for staining. The surface roughness due to wear and chemical damage can also have a negative impact on surface shine followed by an extrinsic staining [37].

#### 2.4.4. Optical Properties

Resin composites are extensively used in restorative dentistry. Optical and structural synchronization of the composite material into the tooth structure and with the neighboring dentition is a significant element for the patient's satisfaction along with acceptance with the dental esthetic restorations. Multiple-layered techniques [38] with resin-based composites of diverse opacity and colors have been experienced to imitate the physical appearance of teeth [39, 40]. So far, this multilayering restorative treatment entails an accurate shade selection along with technically higher skills that frequently raise the working time and cost as well [41]. Therefore, in order to alleviate the treatment intricacy and to improve efficiency, the word “chameleon effect” (blending effect) illustrates the capability of material to attain a shade identical to adjacent tooth structure [42]. This ability of a material has facilitated the recognition of innovative dental composites that makes determination of shades easy. Initially, approach was the so-called “group-shaded” composites that involved an extremely limited shade variety wherein every shade covered a suggested group of VITA classical shades [43]. Currently, the perception of “single-shade” or “one-shade “resin composites was established to explain resin-based composites intended to esthetically imitate every shades with single nominal shade. These resin composites formulated on this broad shade-matching conception, apparently merge flawlessly into the neighboring dentition [41]. Perceived color is determined by the wavelengths reflected from an object [43]. In esthetic restorative materials, for instance, ceramics and resin-based composites, this wavelength reflects as a result of the presence of pigments added as constituents. On the other hand, novel technological methods have introduced the single-shaded resin composites that do not contain pigment, and their optical features are relied upon structural color, a “smart chromatic technology” where the resin-based composite reacts to light waves at a specified frequency by accurately reflecting a particular wavelength within the tooth shade space [44]. Similarly, some research studies [21, 45, 46] demonstrated that the main benefits of OMNICHROMA are based on an enhanced color adjustment potential [47]. One more skill, used to develop Venus Pearl One and Venus Diamond One (Kulzer) that is relied upon “the adaptive light matching” idea, is where the restoration color is attained by absorbing the wavelengths reflected by the adjacent tooth color [48]. Likewise, another study by Brewer et al. proposed that the color stability and optical properties of restorative materials are greatly affected by the changes in the dimensions of filler particles and their composition [49].

Concerning composite translucency, Essentia Universal (GC Europe), Filtek Universal (3 M Oral Care), OMNICHROMA (Tokuyama Dental America, Inc.), SimpliShade Universal Composite (Kerr Corp), and TPH Spectra ST (Dentsply Sirona) are existing in one translucency instead of multiple enamel, dentin, and body shades that have been used to emulate the optical properties of different regions of tooth [46].

#### 2.4.5. Masking Ability of Single-Shade Composite

Though the person eye can perceive the change in color variation, but it is a challenging task to get shade harmonizing towards surrounding tooth structure, particularly in cases of Class III and Class IV restorations or in severely discolored tooth structure where there is no or limited surrounding tooth structure residues [50]. Therefore, in those cases, one-shade resin composite with better opacity is applied as a blocking/masking agent in a thin coat prior to application of smart monochromatic material. This mask assists in camouflaging the inner stained part of the tooth structure and prevents the shade-matching interference due to discoloration. In case of limited surrounding dentition such as large class III and IV restorations, this blocker is placed over the lingual side to lessen shade-matching interference. Additionally, it is valuable in THE reconstruction of an extremely opaque tooth [51].

#### 2.4.6. Mechanical Properties and Curing Depth

Dental restorative materials faced multiple types of stresses such as compressive, tensile, and shear that reflect the mechanical properties of dental filling material [52]. Mechanical properties of dental resin composites can be assessed by determining different aspects including fatigue, hardness, strength, elastic modulus, fracture toughness, edge strength (chipping), and tooth wear [53].

Optimal properties can be achieved by the adequate curing of dental resin composite restorations, whereas inadequate curing causes restoration failure [54]. An insufficiently cured dental resin composite restoration exhibited lower physical and mechanical properties [55]. One of the factors that affect polymerization of dental resin composites is the wavelength of the dental curing light [56].

#### 2.4.7. Wear Resistance and Less Polymerization Shrinkage

Wear resistance is essential for posterior teeth restoration. Occlusal and proximal wear of class II cavities causes failure of posterior composites. It is reported that incidence of failure for both Classes I and II restorations has been predicted to be 40%-50% [57]. High wear resistance for composites leads to improve their longevity, color permanency, and their function; on the other hand, low wear resistance may cause tooth relocation, temporomandibular joint complaints, muscular inflammation, and periodontal infections [58, 59]. Wear of composite is affected by the type and size of filler particles, volumetric ratio, organic matrix nature, and coupling agent. The physical and mechanical properties of dental composites can be improved by modifying new monomers, filler particle size, content change, and filler surface modification [60, 61]. In earlier studies, it was predicted that the wear of older resin composite was about 50-75 µm annually; however, innovative composites have less significant wear which was about 10-20 µm annually [62].

Estelite sigma quick composite comprises uniform silica-zirconia supra-nano spherical-shaped fillers, size of 100-1000 nm with an average size of 200 nm having good wear resistance [63]. Similarly, OMNICHROMA shows an excellent equilibrium among volume loss of the resin composite and human tooth wear. OMNICHROMA is a resin composite that is less prone to damage opposite teeth [44].

#### 2.4.8. Radiopacity

The composition and content of the inorganic filler of composites determine their radiopacity. The radiopacity of a resin rises with the content of high atomic number of fillers. Though, fillers having huge amount of greater atomic number elements apt to have large refractive indices. The radiopacity of smart monochromatic material is moderate and appropriate for diagnostic purposes [64].

## 3. Limitations

- Long-term color stability of smart monochromatic composite in oral cavity is questionable [14].
- The influence of aging on the physical properties of smart monochromatic composite is promising [65].
- The color-matching ability of smart monochromatic composite is excellent with lighter tooth shade while it is not very good with darker tooth shade [66].

## 4. Future Perspective

It could be interesting in the future to test the present material in terms of flexural strength and hardness [67, 68]. Additionally, the mineral deposition should be assessed in order to gain more knowledge about this interesting recently introduced smart monochromatic composite [69].

## 5. Conclusion

Over the past decades of enhancing esthetic restorative materials, there have been many remarkable composite restorations recognized by clinicians worldwide. Nonetheless, smart monochromatic composites are innovative resin composites which presented promising results and most stimulating advancements in the recent times. They are easy to apply, having higher mechanical properties, good wear resistance, and better optical properties along with color stability than those of conventional resin composites that offering admirable esthetics. Further research studies are needed along with follow-up of cases in order to get promising prospects. Additionally, extra experimental trials are also required to document their long-lasting use.

## Figures and Tables

**Table 1:** Various brands of the smart monochromatic composites.

S.No	Smart monochromatic composite	Filler type	Manufacturer	Country	Weblink
1	OMNICHROMA®	Supra-nano fillers	Tokuyama Dental	Japan	https://www.tokuyama-us.com/omnichroma-dental-composite/
2	SpheriChrome	Nano-spherical fillers	Oxford Scientific	Germany	https://oxfordscientificna.com/2022/02/spherichrome-a-new-shade-adaptive-composite-material/
3	Vittra APS unique	Hybrid-nano fillers	FGM Dental Group	Brazil	https://fgmdentalgroup.com/international/aesthetics-products/vittra-aps-unique
4	ONEshade	Microhybrid composite with nanoparticles	Olident	Poland	http://olident.com/en/composites/oneshade-3/
5	CLEARFIL MAJESTY™ ES-2 UNIVERSAL	Nano fillers	Kuraray Noritake Dental Inc	Japan	https://kuraraydental.com/product/clearfil-majesty-es-2-universal/
6	Filtek™ Universal Restorative	Nanofillers, proprietary low-stress monomers, and pigments	3 M	USA	https://www.3m.com/3M/en_US/dental-us/products/restoratives/filtek-universal-restorative/
7	Admira Fusion x-tra®	Nanoparticles with additional microparticles or glass fillers immersed in an ORMOCER matrix.	VOCO	Germany	https://www.voco.dental/us/products/direct-restoration/nano-ormocer/admira-fusion-x-tra.aspx
8	Solare X	Nano fillers, glass fillers and prepolymerised fillers	GC	Japan	https://www.gcindiadental.com/products/composite-restoratives/solare-x/
9	Venus Diamond/Pearl One Shade®	Nanohybrid filler	Kulzer	Germany	https://www.kulzer.com/en/en/products/venus-diamond-pearl-one-shade.html
10	Zen Chroma Universal	Microhybrid and ultrafine radiopaque filler	President Dental	Germany	https://www.presidentdental.com/product_details/PRESIDENT_DENTAL_ZENCHROMA_Universal_Composite/70

## Data Availability

The data supporting this literature review are from previously reported studies and datasets, which have been cited. The processed data are available from the corresponding author upon request.
